# Validation of Six Short and Ultra-short Screening Instruments for Depression for People Living with HIV in Ontario: Results from the Ontario HIV Treatment Network Cohort Study

**DOI:** 10.1371/journal.pone.0142706

**Published:** 2015-11-13

**Authors:** Stephanie K. Y. Choi, Eleanor Boyle, Ann N. Burchell, Sandra Gardner, Evan Collins, Paul Grootendorst, Sean B. Rourke, OHTN Cohort Study Group

**Affiliations:** 1 The Ontario HIV Treatment Network, Toronto, Ontario, Canada; 2 The Institute of Medical Science, Faculty of Medicine, University of Toronto, Toronto, Ontario, Canada; 3 Institute of Sports Science and Clinical Biomechanics, University of Southern Denmark, Odense, Denmark; 4 Dalla Lana School of Public Health, University of Toronto, Toronto, Ontario, Canada; 5 University Health Network, Toronto, Ontario, Canada; 6 Department of Psychiatry, Faculty of Medicine, University of Toronto, Toronto, Ontario, Canada; 7 Division of Social and Administrative Pharmacy, Leslie Dan Faculty of Pharmacy, University of Toronto, Toronto, Ontario, Canada; 8 Department of Economics, McMaster University, Hamilton, Ontario, Canada; 9 St. Michael’s Hospital, Toronto, Ontario, Canada; University of Nebraska Medical Center, UNITED STATES

## Abstract

**Objective:**

Major depression affects up to half of people living with HIV. However, among HIV-positive patients, depression goes unrecognized 60–70% of the time in non-psychiatric settings. We sought to evaluate three screening instruments and their short forms to facilitate the recognition of current depression in HIV-positive patients attending HIV specialty care clinics in Ontario.

**Methods:**

A multi-centre validation study was conducted in Ontario to examine the validity and accuracy of three instruments (the Center for Epidemiologic Depression Scale [CESD_20_], the Kessler Psychological Distress Scale [K_10_], and the Patient Health Questionnaire depression scale [PHQ_9_]) and their short forms (CESD_10_, K_6_, and PHQ_2_) in diagnosing current major depression among 190 HIV-positive patients in Ontario. Results from the three instruments and their short forms were compared to results from the gold standard measured by Mini International Neuropsychiatric Interview (the “M.I.N.I.”).

**Results:**

Overall, the three instruments identified depression with excellent accuracy and validity (area under the curve [AUC]>0.9) and good reliability (Kappa statistics: 0.71–0.79; Cronbach’s alpha: 0.87–0.93). We did not find that the AUCs differed in instrument pairs (p-value>0.09), or between the instruments and their short forms (p-value>0.3). Except for the PHQ_2,_ the instruments showed good-to-excellent sensitivity (0.86–1.0) and specificity (0.81–0.87), excellent negative predictive value (>0.90), and moderate positive predictive value (0.49–0.58) at their optimal cut-points.

**Conclusion:**

Among people in HIV care in Ontario, Canada, the three instruments and their short forms performed equally well and accurately. When further in-depth assessments become available, shorter instruments might find greater clinical acceptance. This could lead to clinical benefits in fast-paced speciality HIV care settings and better management of depression in HIV-positive patients.

## Introduction

Depression affects up to half of people living with HIV [1–4]. However, depression goes unrecognized in about 60–70% of HIV-positive patients in non-psychiatric healthcare settings [5–8]. When depression is left untreated in HIV-positive patients, it can reduce immune activity [9–12] increase the risk of co-morbidities and mortality [13,14], and reduce quality of life [15]. Given the advancements made by highly active antiretroviral therapy (HAART), HIV-positive patients are living longer, and physicians and patients are facing long-term challenges in managing depression [16]. Because of the substantive negative impacts of depression on clinical outcomes normally found among HIV-positive patients, recent guidelines from Canada, U.K. and the U.S. recommend that screening should be undertaken if follow-up in-depth assessments are available [17–19].

Over the past several decades, numerous short and ultra-short screening instruments have been developed to assist in examining depressive symptomatology in non-psychiatric healthcare settings [20,21]. Despite ongoing debates about the effectiveness of these instruments, a recent meta-analysis of 113 studies has shown that most instruments demonstrate adequate performance when used in the initial assessment of depression among patients with physical illness [20].

The 9-item Patient Health Questionnaire (PHQ_9_), the 20-item Center for Epidemiologic Depression Scale (CES-D_20_), and the 10-item Kessler Psychological Distress Scale (K_10_) are three screening instruments commonly used with HIV-positive patients [21,22]. The PHQ_9_ has earned acceptance in primary care and research settings because it is half of the length of most other instruments but maintains comparable sensitivity and specificity [23]. Each item of the PHQ_9_ also corresponds to specific Diagnostic and Statistical Manual of Mental Disorders, 4^th^ edition (DSM-IV) depression diagnosis criteria [23]. The CESD_20_ has the longest history of measuring depression in both HIV-positive patients and the general population [21,22]. It was originally designed for community surveys and has extensively demonstrated its reliability and validity [20,24]. The K_10_ is a short instrument that can broadly screen for both anxiety and depressive disorders [25]. It has strong psychometric properties for distinguishing DSM-IV disorders and its diagnostic accuracy has been shown to have no significant bias by gender or education level [26,27].

Although these three instruments have been extensively evaluated in the general population [24] and in patients with physical illness [20], evaluations of the instruments among HIV-positive patients have been performed mainly in limited-resource settings (i.e., Sub-Saharan Africa) [21,22]. However, the characteristics of the HIV-positive patients in Sub-Saharan Africa—for instance, their literacy levels and their understanding and expression of mental health issues—might be quite different from those of North Americans and affect the evaluation of the instruments. As a result, the psychometric properties of the three instruments and their comparability to a “gold standard” remain unknown for HIV-positive patients in well-resourced settings such as Canada and the United States.

Our multi-centre study sought to determine and compare the diagnostic accuracy and reliability of the three instruments (CESD_20_, K_10_, and PHQ_9_) and their short forms (CESD_10_, K_6_, and PHQ_2_) for current major depression against a gold standard as measured by the Mini International Neuropsychiatric Interview (the “M.I.N.I.”). The study focused on HIV-positive patients receiving HIV primary care in Ontario. Additional study objectives were to determine the optimal cut-points for each screening instrument and to examine potential factors that might affect the diagnostic accuracy of the instruments.

## Materials and Methods

### Study design

We conducted a cross-sectional validation study nested within a larger cohort of participants in HIV care. The Ontario HIV Treatment Network Cohort Study (OCS) is a multi-site, HIV-positive, clinical cohort. Full details regarding the cohort design can be found in a previous publication [28]. Briefly, participants are HIV-positive patients aged 16 years or older receiving care at one of ten specialty HIV clinics in Ontario. Clinical data recorded during the participants’ routine health care visits are abstracted from clinic records and, since 2008, participants have been interviewed annually.

Three OCS sites were included in this validation study: Maple Leaf Medical Centre in Toronto, St. Joseph’s Health Care in London, Ontario, and Windsor Regional Hospital. Participants who agreed to take part in the study received a $20 CAD honorarium. Ethical approval was received from the University of Toronto Human Subjects Review Committee and from the individual study sites (i.e. Ottawa Health Science Network Research Ethics Board, The University of Western Ontario Research Ethics Board for Health Sciences Research involving Human Subjects, St. Michael's Hospital Research Ethics Board, the Research Ethics Board of Health Sciences North, Sunnybrook Health Sciences Centre Research Ethics Board, University Health Network Research Ethics Board, and Windsor Regional Hospital Research Ethics Board). Our consent procedure was approved by all the ethics boards involved and written informed consent was obtained from each participant.

### Recruitment, Data Collection Procedures, and Measures

Between May 1 and December 31, 2014, clinical nurses at each site invited OCS participants to take part in the validation study during their regular appointment. The nurses had received training on how to conduct M.I.N.I. interviews from a psychiatrist specializing in mental disorders and neurocognitive impairments in HIV-positive patients. The nurses were able to consult regularly with the psychiatrist by phone (at the London and Windsor centres) or in person (at the Toronto centre).

Participants completed the three screening instruments (CESD_20_, K_10_, and PHQ_9_). Their short forms (CESD_10_, K_6_, and PHQ_2_) were derived from the long-forms. Details of the three instruments and their short forms are provided in Tables 1 and 2.

**Table 1:** Three Index Screening Instruments.

Twenty-item Center for Epidemiologic Studies Depression Scale (CESD_20_)	Ten-item Kessler Psychological Distress Scale (K_10_)	Nine-item Patient Health Questionnaire (PHQ_9_)
Please tell me how often you have felt the following way during the past week.	During the past month, how often did you feel …	Over the last 2 weeks, how often have you been bothered by any of the following problems?
1. I was bothered by things that usually don’t bother me^a^ ^,^ ^d^	1. … tired out for no good reason?^d^	1. Little interest or pleasure in doing things^c^
2. I did not feel like eating; my appetite was poor^d^	2. …nervous?^b^	2. Feeling down, depressed, or hopeless^c^ ^,^ ^d^
3. I felt that I could not shake off the blues even with help from my family or friends^d^	3. …so nervous that nothing could calm you down?	3. Trouble falling or staying asleep, or sleeping too much^d^
4. I felt I was just as good as other people	4. …hopeless?^b^	4. Feeling tired or having little energy
5. I had trouble keeping my mind on what I was doing^a^ ^,^ ^d^	5. …restless or fidgety?^b^	5. Poor appetite or overeating^d^
6. I felt depressed^a^ ^,^ ^d^	6. …so restless that you could not sit still?	6. Feeling bad about yourself—or that you are a failure or have let yourself or your family down
7. I felt that everything I did was an effort^a^	7. …depressed?^d^	7. Trouble concentrating on things, such as reading the newspaper or watching television^d^
8. I felt hopeful about the future^a^	8. …so depressed that nothing could cheer you up?^b^	8. Moving or speaking so slowly that other people could have noticed. Or the opposite—being so fidgety or restless that you have been moving around a lot more than usual
9. I thought my life had been a failure	9. …that everything was an effort?^b^	9. Thoughts that you would be better off dead or of hurting yourself in some way.
10. I felt fearful^a^	10. …worthless?^b^	
11. My sleep was restless^a^ ^,^ ^d^		
12. I was happy^a^		
13. I talked less than usual		
14. I felt lonely^a^		
15. People were unfriendly		
16. I enjoyed life		
17. I had crying spells		
18. I felt sad		
19. I felt that people dislike me		
20. I could not get “going” ^a^ ^,^ ^d^		

^a^ Ten items are in the CESD_10_, a short-form of CESD_20._

^b^ Six items are in the K_6_, a short-form of K_10._

^c^ Two items are in the PHQ_9_, a short-form of PHQ_2._

^d^ These items correspond to previously reported somatic symptoms of HIV infection (Kalichman, Rompa, &Cage, 2000).

**Table 2:** Summary of Properties for Three Index Screening Instruments.

	Index Instruments		
	Center for Epidemiologic Studies Depression Scale (CESD_20_)	Kessler Psychological Distress Scale (K_10_)	Patient Health Questionnaire (PHQ_9_)
**Time frame**	Past week	Past month	Past two weeks
**Source**	Radloff (1977)	Kessler et al. (2002)	Spitzer et al. (1994)
**Duration**	4–5 minutes	2–3 minutes	2–4 minutes
**Number of questions**	20	10	9
**Which condition(s) is screen designed to measure?**	Major depression	Depression and anxiety disorder	Major depression
**Derived short form**	CESD_10_ (10 items)	K_6_ (6 items)	PHQ_2_ (2 items)
**Measurement Scale**	4-point Likert scale	5-point Likert scale	4-point Likert scale
	*1*.*Rarely or none of the time (less than 1 day)*	*1*. *None of the time*	*1*.*Not at all*
	*2*. *Some or a little of the time (1–2 days)*	*2*. *little of the time*	*2*.*Several days*
	*3*. *Occasionally or a moderate amount of time (3–4 days)*	*3*. *Some of the time*	*3*. *More than half the day*
	*4*. *Most or all of the time (5–7 days)*	*4*. *Most of the time*	*4*. *Nearly every day*
		*5*. *All of the time*	
**Score format**	Original form:	Original form:	Original form:
	*1*. *Total score*: *0–60*	*1*. *Total score*: *10–50*	*1*. *Total score*:*0–27*
	*2*. *Per item score*: *0–3*	*2*. *Per item score*: *1–5*	*2*. *Per item score*: *0–3*
	Short form:	Short form:	Short form:
	*1*. *Total score*: *0–30*	*1*. *Total score*: *6–30*	*1*. *Total score*:*0–6*
	*2*. *Per item score*: *0–3*	*2*. *Per item score*: *1–5*	*2*. *Per item score*:*0–3*
	A high total score indicates more depressive symptoms	A high total score indicates more depressive and anxiety symptoms	A high total score indicates more depressive symptoms
**Is the instrument based on DSM criteria?**	No	No	Yes^a^
**Can the instrument distinguish the severity level of depression?**	Yes	Yes	Yes
**Possible cut-offs**	For general population or patients with physical illnesses: *ranged from 16 to 27* ^b^	For general population or patients with physical illnesses: *ranged from 18 to 35* ^*c*^	For general population or patients with physical illnesses: *ranged from 8 to 11* ^*d*^
	For HIV-positive patients: *ranged from 16 to 22* ^*e*^	For HIV-positive patients: *ranged from 18 to 28* ^*f*^	For HIV-positive patients: *ranged from* cut-offs ≥10^g^
**Performance Statistics**	For general population or patients with physical illnesses:	For general population or patients with physical illnesses:	For general population or patients with physical illnesses:
	*1*. *Area under the curve (AUC) ranged from 0*.*78 to 0*.*96* ^*b*^ ^,^ ^*c*^ ^,^ ^*j*^	*1*. *Area under the curve (AUC) ranged from 0*.*87 to 0*.*93* ^*d*^ ^,^ ^*g*^ ^,^ ^*k*^	*1*. *Area under the curve (AUC) ranged from 0*.*78 to 0*.*91* ^*c*^ ^,^ ^*e*^ ^,^ ^*f*^ ^,^ ^*j*^
	*2*. *At optimal cut-offs*, *sensitivity ranged from 0*.*56 to 0*.*95*, *specificity ranged from 0*.*76 to 0*.*85*, *positive predictive value ranged from 0*.*11 to 0*.*82*, *negative predictive value ranged from 0*.*75 to 0*.*99* ^*b*^ ^,^ ^*c*^ ^,^ ^*j*^	*2*. *At optimal cut-offs*, *sensitivity ranged from 0*.*73 to 1*.*0*, *and specificity ranged from 0*.*34 to 0*.*90* ^*d*^ ^,^ ^*l*^	*2*. *At optimal cut-offs*, *sensitivity ranged from 0*.*76 to 0*.*88*, *specificity ranged from 0*.*72 to 0*.*88*, *positive predictive value ranged from 0*.*18 to 0*.*92*, *negative predictive value ranged from 0*.*95 to 0*.*98* ^*c*^ ^,^ ^*e*^ ^,^ ^*f*^ ^,^ ^*j*^
	*3*. *Internal consistency (Cronbach’sα) ranged from 0*.*85 to 0*.*90* ^*b*^	*3*. *Internal consistency (Cronbach’s*α*) ranged from 0*.*90 to 0*.*93* ^*d*^ ^,^ ^*g*^ ^,^ ^*k*^	*3*. *Internal consistency (Cronbach’s*α*) ranged from 0*.*86 to 0*.*89* ^*c*^ ^,^ ^*j*^
	*4*. *Test-and-retest reliability ranged from 0*.*45 to 0*.*70* ^*b*^		*4*. *Test-and-retest reliability was 0*.*84* ^*c*^ ^,^ ^*j*^
	For HIV-positive patients:	For HIV-positive patients:	For HIV-positive patients:
	*1*. *Area under the curve (AUC) ranged from 0*.*76 to 0*.*94* ^*h*^ ^,^ ^*i*^	*1*. *Area under the curve (AUC) ranged from 0*.*77 to 0*.*82* ^*h*^ ^,^ ^*i*^ ^,^ ^*l*^	*1*. *Area under the curve (AUC) ranged from 0*.*87 to 0*.*96* ^*h*^ ^,^ ^*I*^ ^m^ ^,^ ^*n*^
	*2*. *At optimal cut-offs*, *sensitivity ranged from 0*.*73 to 0*.*87 and specificity ranged from 0*.*44 to 0*.*80* ^*h*^ ^,^ ^*i*^	*2*. *At optimal cut-offs*, *sensitivity ranged from 0*.*67 to 0*.*83*, *specificity ranged from 0*.*72 to 0*.*77*, *positive predictive value was 0*.*29*, *negative predictive value ranged was 0*.*94* ^*h*^ ^,^ ^*i*^ ^,^ ^*l*^	*2*. *At optimal cut-offs*, *sensitivity ranged from 0*.*27 to 0*.*91*, *and specificity ranged from 0*.*83 to 0*.*94* ^*h*^ ^,^ ^*i*^ ^,^ ^m^ ^,^ ^*n*^
	*3*. *Internal consistency (Cronbach’s α) ranged from 0*.*84 to 0*.*90* ^*i*^	*3*. *Internal consistency (Cronbach’s α) ranged from 0*.*8* ^*i*^	*3*. *Internal consistency (Cronbach’s* α*) ranged from 0*.*73 to 0*.*93* ^*i*^ ^,^ ^*n*^

^a^ The PHQ-9 includes a DSM-IV algorithm to generate a diagnosis of major depression but it does not include DSM-IV exclusion criteria for excluding the condition.

^b^ Source: Radloff (1977)

^c^ Source: Meader (2011)

^d^ Source: Kessler et al. (2002)

^e^ Source: Spitzer et al. (1994)

^f^ Source: Kroenke et al. (2001)

^g^ Furukawa, Kessler, Slade, & Andrews (2003)

^h^ Source: Akena et al. (2013)

^i^ Source: Tsai (2014)

^j^ Source: National Institute for Health and Care Excellence (2009)

^k^ Source: Cairney et al. (2007)

^l^ Source: Spies et al.(2009)

^m^ Source: Pence et al.(2012)

^n^ Source: Monahan et al. (2009)

Following the completion of the M.I.N.I. interview, and on the same date, the nurses administered an electronic version of the M.I.N.I. [29] to diagnose current major depressive disorder. The M.I.N.I. is a short and widely adopted structured interview that takes about 15 minutes to complete and can be easily administered by a lay interviewer [29]. The M.I.N.I. has high sensitivity (94–96%) and specificity (79–88%) for identifying major depressive disorder when compared to the structured clinical interviews for the DSM-IV (SCID) and the International Classification of Disease, 10^th^ revision (ICD-10) criteria [29–31]. Nurses and participants were blinded to the results of the M.I.N.I. interviews.

### Covariates

We also assessed whether certain characteristics of patients might affect the diagnostic accuracy of the screening instruments. Patient information was obtained through interviews administered by the nurses on the study date or during a previous appointment [28]. Measurement details for key characteristics are provided in Table 3.

**Table 3:** Description of Individual Covariates of the Sample.

Variables	Categories	Measuring Instruments
Age	Continuous variable	Derived from birth date and interview date
Sex	Male, female	Self-reported
Immigrant	Yes or No	Self-reported as not Canadian-born
Annual household income below $20,000 CAD	Yes or No	Self-reported
Completion of high school or less	Yes or No	Self-reported
Recipient of Ontario Disability Support Program (ODSP) subsidies	Yes or No	Self-reported. The ODSP is a provincial social assistance program that provides income support for Ontario residents who have a financial need and who have substantial mental or physical impairment for a year or more that has been verified by an approved health care professional. The impairment must restrict the individual’s ability to work, to take care of themselves and/or to take part in their community life.
Current smoker	Yes or No	Self-reported
Harmful alcohol consumption (in past 12 months)	Yes or No	Harmful alcohol consumption in past 12 months was assessed using 3-item Alcohol Use Disorders Identification Test (AUDIT-C) instrument (male: cut-point≥4; female: cut-point≥3)^a^ ^,^ ^b^
CD4 cell count (in past 6 months)	Yes or No	Yes if CD4 cell counts less than 200µL during past 6 months
Non-suppressed viral load (in past 6 months)	Yes or No	Yes if non-suppressed viral loads (>50µL) during past 6 months
Years since HIV diagnosis	Continuous variable	Derived from the interview date and the date of HIV diagnosis

^a^ Source: Bush K. (1998)

^b^ Source: Bradley KA et al. (2003)

### Statistical Analysis

After the data were collected and de-identified, results from the M.I.N.I. diagnoses and total scores for the three screening instruments were generated at the OCS office by the lead investigator (S. C.) who was independent to the data collection. Our statistical analysis plan was four-fold: 1) To examine the diagnostic accuracy of the three screening instruments and their short forms; 2) To identify optimal cut-points for the screening instruments; 3) To examine the effects of seven previously documented somatic symptoms of HIV infection [32] on the diagnostic accuracy and performance of the screening instruments; and 4) To examine inter-rater agreement for pairs of the three instruments and internal consistency of each instrument.

We first used descriptive statistics to describe baseline characteristics, scores of the screening instruments and their short forms, and the prevalence of DSM-IV defined psychiatric disorders among study participants. We also assessed the differences by age (Student’s t-test) and by sex (Pearson’s chi-squared test) between our sample and the rest of the OCS participants who are currently active in the OCS.

We then used non-parametric crude and adjusted Receiver Operating Characteristic (ROC) analyses to examine the criterion validity and accuracy of the three screening instruments and their short forms as compared to the M.I.N.I. First, overall psychometric property of each instrument was described by a global measure: area under the ROC curve (AUC). In general, values of AUC (ranged: 0.5 to 1) greater than 0.8 and 0.9 indicated either good or excellent performance respectively. Second, we used non-parametric Mann-Whitney U-test to assess for equality of ROC curves of the instruments [33]. For each screening instrument, several criterion validity statistics were reported at each pre-defined cut-point: sensitivity (Se), specificity (Sp), positive predictive value (PPV), negative predictive value (NPV), positive likelihood ratio (LR+), and negative likelihood ratio (LR-). Finally, adjusted multivariable non-parametric ROC analyses were performed [34] because some covariates may have an impact on the accuracy of the instruments. Bivariate analyses were first performed to examine crude associations between the ROC curve of each instrument and each covariate. Covariates with a p-value<0.25 were entered into the final multivariable model [35]. Coefficients of the adjusted multivariable model generally reflect the impact of a specific covariate on the adjusted ROC curve by assuming a linear relationship exists between diagnostic accuracy of the instruments and each covariate. A value of zero indicates no effect. We also assessed the overall impact of the covariates by comparing crude and adjusted AUCs for each instrument.

There are many criteria for determining optimal cut-points for screening instruments [36–40]. In our study, we adopted three common criteria: Youden index (YI) [defined as Se+Sp-1] [41], distance (PROC01) between the optimal point on the ROC curve and the point of (0, 1), which is an ideal point corresponding to a sensitivity and specificity equal to 1 [defined as (NPV-1)^2^+(PPV-1)^2^] [37,39], and diagnostic odds ratios (DOR) [defined as LR+/LR-] [40,42]. The YI (ranged:-1 to 1) is a single index that balances the sensitivity and specificity where the greater its value, the better the validity of the cut-point. The PROC01 (ranged: 0 to 2) is a single index that is balanced on both the NPV and PPV and its minimum value indicates the best validity for the cut-point. The DOR (ranged: 0 to infinity) is a summary statistic that indicates the odds for a patient to have a positive result in the screening for depression when compared to a non-diseased patient. The greater the value of the DOR (ranged: 0 to infinity) indicates a better predictive performance. Because we were evaluating the predictive performance of each screening instrument, we made our final decision on the optimal cut-point based on the following order: DOR, PROC01, and YI.

We further examined the diagnostic accuracy of the screening instruments by removing some items (i.e., fatigue, sleep, appetite, not being able to shake the blues, feeling bothered, feeling depressed, and lack of concentration) from the instruments that have been previously reported as somatic symptoms of HIV. It is possible that these items might inflate depression scores [32]. For each instrument, we repeated the adjusted ROC analysis with items related to the somatic symptoms removed. We then used Wald test to determine for the equality between the adjusted ROC curves of the original instruments and their corresponding reduced scales. The standard error of the hypothesis test was obtained from a bias-corrected bootstrap method [43,44].

Finally, we used Cohen’s Kappa statistic (ranged: -1 to 1; 0.6–0.7, 0.8–0.9 and >0.9 representing good, very good and excellent agreement, respectively) to examine the inter-rater agreement of each instrument pair by dichotomizing total scores of the instrument at the optimal cut-points. Cronbach’s alpha (ranged: 0 to 1; 0.7–0.9 and >0.9 representing good and excellent consistency, respectively) was used to examine internal consistency of the instruments.

All reported 95% confidence intervals were constructed by bias-corrected bootstrap method with 2000 replicates [45]. All statistical analyses were 2-sided with statistical significance defined as a p-value less than 0.05 and were performed by using STATA IC v.13.1 [46].

### Sample size calculation

Based on two receiver operating characteristics (ROC) curves power analysis, we would have required 177 individuals with complete data to achieve an 80% statistical power (assuming a prevalence of 17% and a difference of 0.15 in AUC to be detected between two ROC curves) [47,48].

## Results

Two hundred and thirty-seven HIV-positive patients (aged ≥ 18 years) agreed to participate in the validation study. When we compared the characteristics of the validation study participants to the remainder of the cohort, we found that participants were slightly younger (mean age: 47 v. 51 years; p-value: 0.02) and more likely to be male (86 v. 82%; p-value: 0.08).

Of the 237 HIV-positive patients initially included, we excluded 47 participants on the basis of information missing from either the M.I.N.I. or one of the screening instruments. Our final analytical sample was 190 patients. Of these, 179 had provided demographic, psychosocial, and behavioural information during a regular OCS interview conducted before the validation study began.

### Prevalence of Depression and Characteristics of the Sample

Table 4 presents baseline characteristics and the prevalence of DSM-defined psychiatric disorders of the sample. Of the 179 patients who provided demographic information, the mean age was 47 (SD = 11) years and 87% were male. Based on DSM-IV criteria from the M.I.N.I., twenty-nine patients (16%) were identified with current major depression within the past two weeks. The mean and standard derivation of distribution of total scores of the CESD_20_, K_10_, PHQ_9_, CESD_10_, K_6_, and PHQ_2_ were 14(13), 18(8), 5(5), 8(7), 11(5), and 1(2) respectively. About half of the HIV-positive patients reported annual household incomes of less than $20,000 CAD and about half were recipients of Ontario Disability Support Program subsidies. About 40% of patients had at least one of the nine psychiatric disorders that we examined.

**Table 4:** Baseline Characteristics, the Mean Scores of the Screening Instruments the Sample, and the Prevalence of DSM-defined Psychiatric Disorders of the Sample (N = 179^a^).

Characteristics	Total	
	(N = 179^a^)	
***Baseline Characteristics***		
Age, mean (SD^b^)	47	(11)
Male	156	(87%)
Annual household income < $20K CAD	89	(50%)
Immigrant^c^	45	(25%)
Receipt of Ontario Disability Support Program subsidies ^d^	80	(45%)
Completed high school or less	49	(27%)
Recreational drug use (in past 6 months)^e^	42	(23%)
Current smokers	70	(39%)
Harmful alcohol consumption^f^	66	(37%)
CD4 cell count < 350 µL(in past 6 months)	7	(4%)
Viral loads ≤ 50 µL (in past 6 months)	150	(84%)
Years since HIV diagnosis, mean (SD^b^)	14	(8)
***Results of Three Screening Instruments for Depressive Disorder and their Short Forms*, *mean (SD*** ^***b***^ ***)***		
20-item Center for Epidemiologic Studies Depression Scale (CESD_20_)	14	(13)
10-item Kessler Psychological Distress Scale (K_10_)	18	(8)
9-item Patient Health Questionnaire (PHQ_9_)	5	(5)
10-item Center for Epidemiologic Studies Depression Scale (CESD_10_)	8	(7)
6-item Kessler Psychological Distress Scale (K_6_)	11	(5)
2-item Patient Health Questionnaire (PHQ_2_)	1	(2)
***Psychiatric disorders (defined by Mini International Neuropsychiatric Interviews [M*.*I*.*N*.*I*.*]*** ^***g***^		
Major Depressive Disorder (single episode), past two weeks	29	(16%)
Bipolar disorder, past month	10	(6%)
Posttraumatic stress disorder, past month	13	(7%)
Alcohol dependence, past year	16	(9%)
Alcohol abuse, past year	8	(6%)
Drug dependence, past year	28	(16%)
Drug abuse, past year	18	(10%)
Generalized anxiety disorder, past 6 months	23	(13%)
≥ 1 psychiatric disorders	70	(39%)

^a^ Of 190 patients, 179 provided demographic, psychosocial and behavioural information.

^b^ SD = Standard Derivation

^c^ Immigrants are study participants who are not Canadian-born.

^d^ Receipt of Ontario Disability Support Program subsidies served as a proxy for physical or mental disability.

^e^ Recreational drug use was defined as use of drugs either not prescribed or not used according to instructions.

^f^ Harmful alcohol consumption in past 12 months was assessed using the 3-item Alcohol Use Disorders identification Test. (AUDIT-C) instrument (male: cut-point: ≥ 4; female: cut-point: ≥3) by Bush et al. (1998) and Bradley et al. (2003). AUDIT-C is an ultra-brief assessment developed by World Health Organization (WHO) to examine excess consumption of alcohol.

^g^ Frequency and proportion for dysthymia (recurrent depression) was not reported because cell size was <6.

### Overall Psychometric Properties and Criterion Validity from ROC Analysis

**Fig 1:**
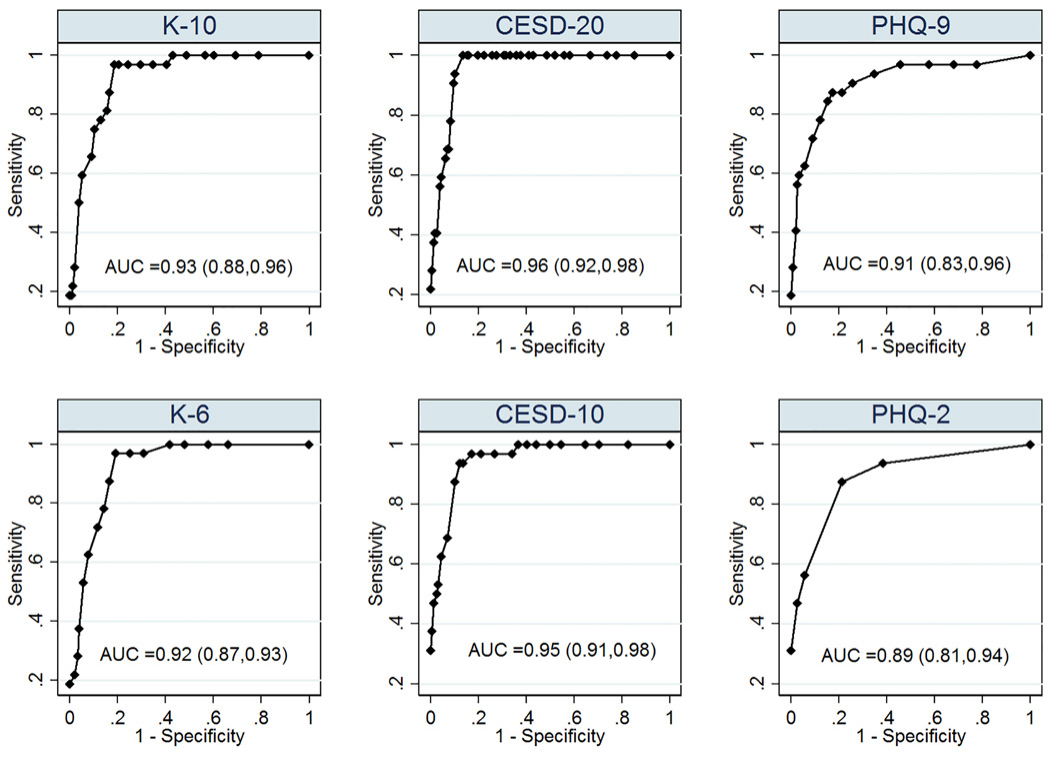
Crude ROC Curves of the Index Screening Instruments and their Short Forms for Current Major Depression (N = 190); Footnotes: All reported 95% confidence intervals were constructed by bias-corrected bootstrap method with 2000 replicates (Efron & Tibshirani, 1994)

Fig 1 presents the unadjusted non-parametric AUCs of the screening instruments and their short forms against the M.I.N.I. Overall, we found that all of the instruments were able to discriminate current major depression with excellent accuracy and validity (AUC >0.9). We estimated that AUCs of CESD_20,_ K_10_, and PHQ_9_ were approximately 0.96 (95% CI: 0.92, 0.98), 0.93 (95% CI: 0.88, 0.96) and 0.91 (95% CI: 0.83, 0.96) respectively. Their short forms performed comparably: CESD_10_ (AUC: 0.95; 95% CI: 0.91, 0.98), K_6_ (AUC: 0.92; 95% CI: 0.87, 0.95), and PHQ_2_ (AUC: 0.89; 95% CI: 0.81, 0.94). We did not find that the AUCs were significantly different between each pair of instruments (e.g. absolute value of [AUC_CESD-20_-AUC_PHQ-9_ = 0.05], p-value>0.1) or between the instruments and their corresponding short forms (e.g. absolute value of [AUC _PHQ-9_-AUC_PHQ-2_] = 0.02, p-value >0.3) (Table 5).

**Table 5:** Comparison of AUCs between Pairs of Index Screening Instruments and the AUCs between Original and the Short-form of Each Instrument (N = 190).

Comparisons	Crude AUC_1_ ^a^ (95% CI^c^)	Crude AUC_2_ ^b^ (95% CI^c^)	|AUC_1_ –AUC_2_|^d^	P-value^e^
***Pairs of Index Screening Instruments***				
CESD_20_ and K_10_	0.96 (0.92, 0.98)	0.93 (0.88, 0.96)	0.03	0.09
CESD_20_ and PHQ_9_	0.96 (0.92, 0.98)	0.91 (0.83, 0.96)	0.05	0.1
K_10_ and PHQ_9_	0.93 (0.88, 0.96)	0.91 (0.83, 0.96)	0.02	0.6
***Pairs between Original and its short Forms***				
CESD_20_ and CESD_10_	0.96 (0.92, 0.98)	0.95 (0.91, 0.98)	0.01	0.6
K_10_ and K_6_	0.93 (0.88, 0.96)	0.92 (0.87, 0.93)	0.01	0.5
PHQ_9_ and PHQ_2_	0.91 (0.83, 0.96)	0.89 (0.81, 0.94)	0.02	0.3

* p<0.05 ** p<0.01 ***p<0.001

^a^ AUC_1_ was defined as the area under the curve of the first index screening instrument of the specific pair.

^b^ AUC_2_ was defined as the area under the curve of the second index screening instrument of the specific pair.

^c^ CI = confidence interview. All the reported 95% confidence intervals were constructed by bias-corrected bootstrap method with 2000 replicates (Efron & Tibshirani, 1994).

^d^ |AUC_1_ –AUC_2_| was defined as an absolute value of difference between AUCs of the specific comparison pair.

^e^ Mann-Whitney U- test was used to assess for equality of AUCs of the specific pair (E.R. DeLong, D.M. DeLong, & Clarke-Pearson, 1988).

**Fig 2:**
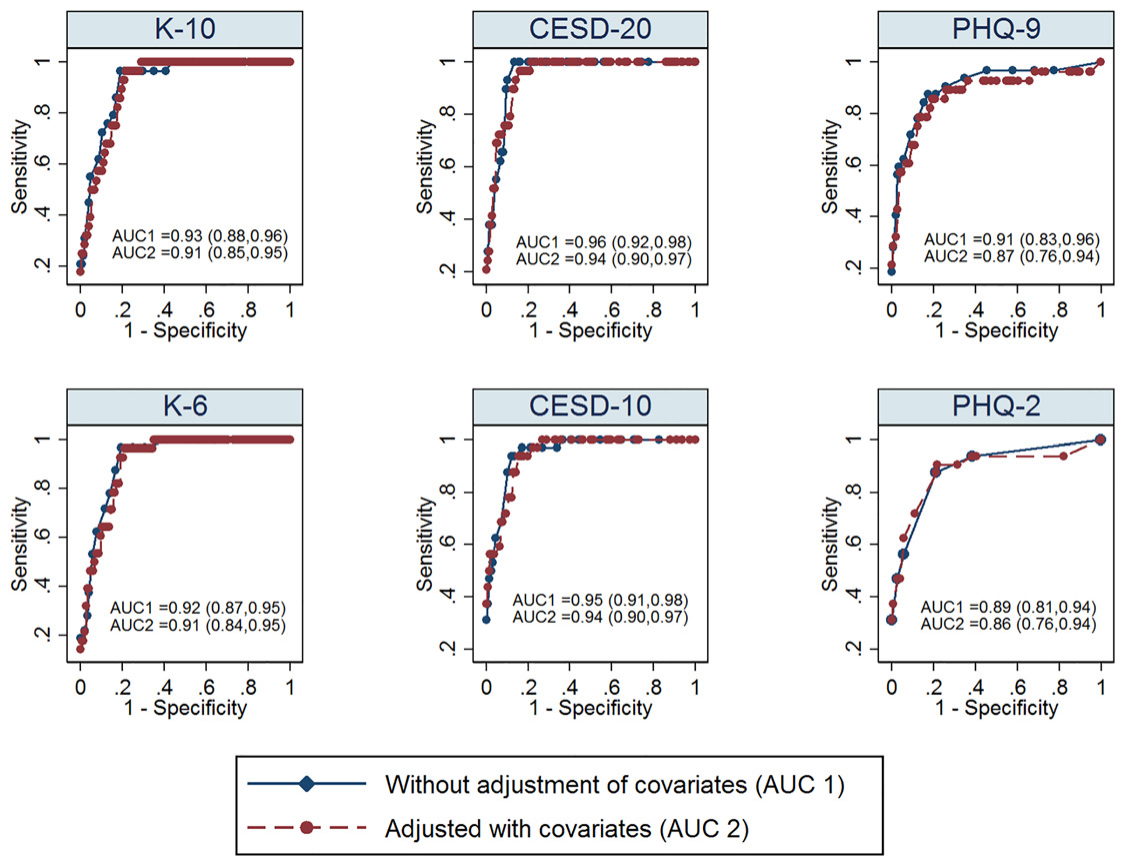
Adjusted ROC Curves of the Index Screening Instruments and their Short Forms for Major Depressive Disorder (N = 179^a^); Footnotes: All reported 95% confidence intervals were constructed by bias-corrected bootstrap method with 2000 replicates (Efron & Tibshirani, 1994); AUC = Area under the curve; ^a^Of 190 patients, 179 provided demographic, psychosocial and behavioural information;

Of the 179 patients who provided demographic information, our multivariable ROC analysis indicated that the receipt of Ontario Disability Support Program subsidies might make discriminatory ability of these instruments weaker for CESD_10_ and PHQ_9_ (Table 6). Additionally, though the ROC curves and AUCs after controlling for covariates were similar to those without the adjustment, there were differences between the crude and adjusted ROC curves for each instrument (Fig 2)

**Table 6:** Multivariable ROC Analysis^a^ for the Index Screening Instruments and their Short Forms for Current Major Depression Disorder (N = 179^b^).

Covariates	CESD_20_	K_10_	PHQ_9_	CESD_10_	K_6_	PHQ_2_
	β^c^ (95% CI)	β ^c^ (95% CI)	β ^c^ (95% CI)	β ^c^ (95% CI)	β ^c^ (95% CI)	β ^c^ (95% CI)
Receipt of Ontario Disability Support Program subsidies ^d^	-0.1(-1.0, 0.8)	-0.5(-1.5, 0.5)	**-0.9** * **(-1.7, -0.3)**	**-0.9** * **(-1.8, -0.1)**	0.1(-0.9, 1.0)	-0.6(-1.9, 0.7)
Male	0.2(-1.5, 1.9)	-0.9(-2.4, 0.3)	-0.5(-1.9, 0.8)	0.9(-0.4, 2.3)	-0.8(-2.2, 0.6)	**-1.5** * **(-2.6, -0.5)**

* p<0.05 ** p<0.01 ***p<0.001

^a^ Adjusted multivariable non-parametric ROC analyses were also performed (Jane H., et al., 2009) because some covariates may affect the accuracy of the instruments. Bivariate analysis was first performed to examine the crude association between the ROC curve of each instrument and each covariate. Covariates generally entered into the final multivariable model if p-value <0.25 in bivariate analysis (Vittinghoff E., et al., 2005). The multivariable models also controlled for other covariates (age, current smoking status, immigration status, educational attainment, recent CD4 cell count, and recent viral loads), but not all of them were statistically significant.

^b^ Of 190 patients, 179 provided demographic, psychosocial and behavioural information.

^c^ Coefficients of adjusted multivariable model generally reflect impacts of a specific covariate on the adjusted ROC curve by assuming a linear relationship (Jane H., et al., 2009).

^d^ Receipt of Ontario Disability Support Program (ODSP) subsidies was used as a proxy measure for physical or mental disability

### Optimal Cut-points

Table 7 presents results for the diagnostic accuracy of the instruments at a range of possible cut-points evaluated in prior studies. Based on the best results for DOR, PROC01, and YI, we identified optimal cut-points of 22 (Se:0.97;Sp:0.81) for K_10_, 23 (Se:1.0;Sp:0.87) for CESD_20_, 8 (Se:0.86;Sp:0.82) for PHQ_9_, 13 (Se:0.97;Sp:0.81) for K_6_, 12 (Se:0.97;Sp:0.82) for CESD_10,_ and 4 (Se:0.45;Sp:0.97) for PHQ_2_ respectively. Except for PHQ_2_, these instruments showed an excellent NPV (>0.90) for ruling-out major depression, but moderate PPV (0.49–0.51) for ruling-in the condition at their optimal cut-points. Although PHQ_2_ showed moderate PPV (0.7), its sensitivity was poor (0.45); hence, it was likely to miss some depression cases.

**Table 7:** Diagnostic Accuracy of the Index Screening Instruments and their Short Forms by Cut-offs for Current Major Depression (N = 190).

Cut-off	Sensitivity	Specificity	PPV	NPV	Correctly Classified (%)	LR+	LR-	PROC01^a^	Youden index^b^	Diagnostic Odds Ratio^c^
**K**_**10**_										
≥18	0.97	0.65	0.35	0.99	69.83	2.73	0.053	0.43	0.62	54
≥20	0.97	0.75	0.42	0.99	78.21	3.81	0.046	0.33	0.72	76
≥21	0.97	0.79	0.47	0.99	82.12	4.67	0.044	0.28	0.76	117.5
**≥22**	**0.97**	**0.81**	**0.49**	**0.99**	**83.80**	**5.17**	**0.042**	**0.26**	**0.78**	**130**
≥24	0.79	0.85	0.50	0.96	83.80	5.17	0.24	0.25	0.64	26
≥26	0.72	0.90	0.58	0.94	87.15	7.24	0.31	0.18	0.62	24
≥28	0.55	0.95	0.68	0.92	88.80	11.82	0.47	0.11	0.5	23.6
**CESD**_**20**_										
≥16	1.0	0.72	0.40	1.00	76.54	3.57	0.00	0.35	0.72	∞
≥18	1.0	0.77	0.46	1.00	81.01	4.41	0.00	0.30	0.77	∞
≥20	1.0	0.84	0.52	1.00	86.59	6.25	0.00	0.21	0.84	∞
≥22	1.0	0.85	0.54	1.00	87.15	6.52	0.00	0.19	0.85	∞
**≥23**	**1.0**	**0.87**	**0.58**	**1.00**	**88.83**	**7.50**	**0.00**	**0.16**	**0.87**	∞
≥24	0.93	0.90	0.65	0.99	90.50	9.31	0.077	0.13	0.83	116
≥26	0.76	0.91	0.62	0.95	88.83	8.75	0.26	0.15	0.67	34
≥28	0.66	0.92	0.62	0.95	87.71	8.19	0.37	0.16	0.58	22
**PHQ**_**9**_										
**≥8**	**0.86**	**0.82**	**0.48**	**0.97**	**82.68**	**4.79**	**0.17**	**0.28**	**0.68**	**28**
≥9	0.83	0.85	0.51	0.96	84.36	5.40	0.20	0.24	0.68	27
≥10	0.76	0.88	0.55	0.95	86.03	6.32	0.27	0.21	0.64	23
≥11	0.69	0.91	0.59	0.94	87.15	7.39	0.34	0.17	0.60	22
**K**_**6**_										
≥11	0.97	0.69	0.37	0.99	73.18	3.08	0.05	0.39	0.66	62
**≥13**	**0.97**	**0.81**	**0.49**	**0.99**	**83.24**	**4.99**	**0.04**	**0.26**	**0.78**	**125**
≥15	0.76	0.86	0.51	0.95	84.36	5.41	0.28	0.24	0.62	19
≥17	0.59	0.93	0.62	0.92	87.15	8.00	0.45	0.15	0.52	18
**CESD**_**10**_										
≥9	0.97	0.65	0.35	0.99	70.39	2.79	0.05	0.43	0.62	56
≥10	0.97	0.73	0.41	0.99	76.54	3.53	0.05	0.35	0.7	71
≥11	0.97	0.79	0.47	0.99	81.56	4.53	0.04	0.28	0.76	113
**≥12**	**0.97**	**0.82**	**0.51**	**0.99**	**84.36**	**5.36**	**0.04**	**0.24**	**0.79**	**134**
**PHQ**_**2**_										
≥2	0.86	0.79	0.44	0.97	79.89	4.04	0.18	0.32	0.65	22
≥3	0.55	0.94	0.64	0.92	87.71	9.20	0.47	0.14	0.49	20
**≥4**	**0.45**	**0.97**	**0.74**	**0.90**	**88.83**	**16.8**	**0.57**	**0.08**	**0.42**	**29**

^a^ PROC01 is defined as a distance between the ROC curve and the point of (0, 1) [defined as (NPV-1)^2^ + (PPV-1)^2^] (Gallop et al, 2003; Vermont et al, 1991).

^b^ Youden index (YI) is defined as Se+Sp-1 (Youden, 1950).

^c^ Diagnostic odds ratios (DOR) is defined as LR+/LR- (Böhning et al., 2011; Glas et al., 2003).

### Impacts of Somatic Symptoms of HIV Infection on Diagnostic Accuracy

**Fig 3:**
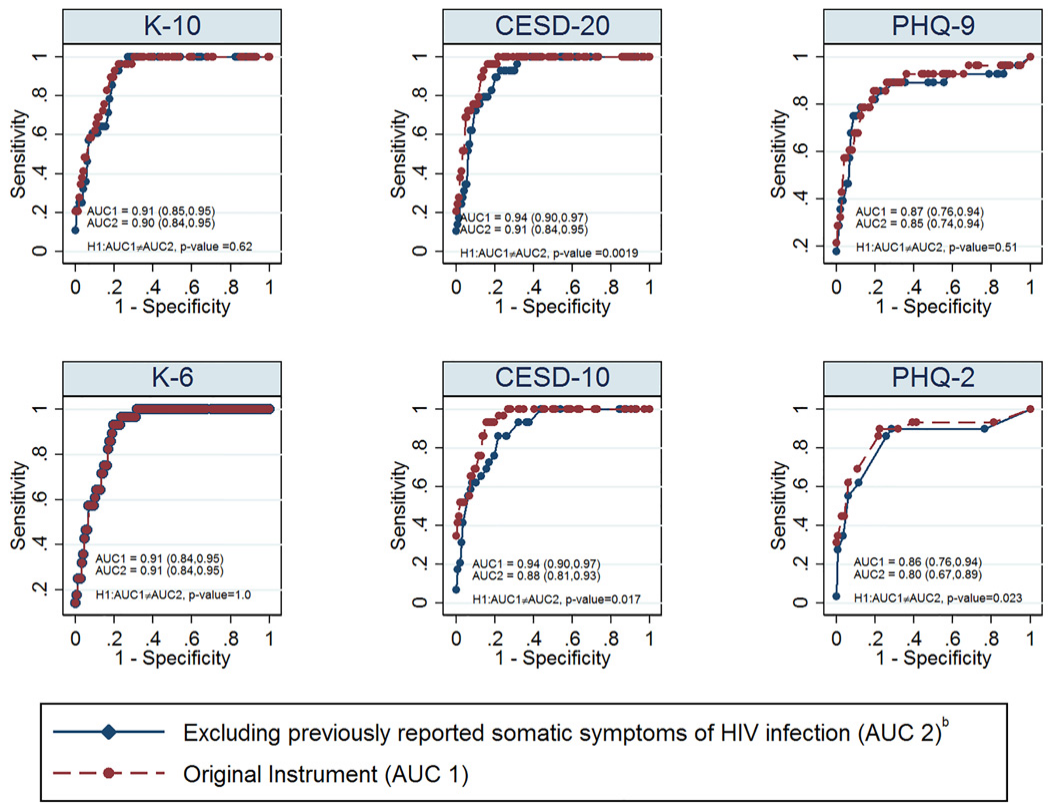
Comparison Between Adjusted ROC Curves of the Original Instruments for Current Major Depression and that of their Corresponding Reduced Scales After Removing Items Related to Somatic Symptoms of HIV (N = 179^a^); Footnotes: All reported 95% confidence intervals were constructed by bias-corrected bootstrap method with 2000 replicates (Efron & Tibshirani, 1994); AUC = Area under the curve; ^a^Of 190 patients, 179 provided demographic, psychosocial and behavioural information; ^b^Items (i.e., fatigue, sleep, appetite, not being able to shake the blues, feeling bothered, feeling depressed, and lack of concentration) correspond to previously reported somatic symptoms of HIV infection (Kalichman, Rompa, &Cage, 2000).

When we removed items (i.e., fatigue, sleep, appetite, not being able to shake the blues, feeling bothered, feeling depressed, and lack of concentration) [32] that were previously reported as somatic symptoms of HIV infection from the original screening instruments and their short forms for current major depression, we found that the results of adjusted AUCs of CESD_20_ (p-value = 0.0019), CESD_10_ (p-value = 0.017) and PHQ_2_ (p-value = 0.023) were significantly reduced (Fig 3).

### Reliability

Table 8 presents the results of inter-rater agreement of pairs of the three instruments and internal consistency for each instrument. Each pair of the three instruments demonstrated good inter-rater agreement (Cohen’s Kappa statistics: 0.71–0.79). The instruments also showed good-to-excellent internal consistency (Cronbach’s alpha: 0.87–0.93)

**Table 8:** Inter-rater Agreement of Pairs of Index Screening Instruments and Internal Consistency for each Instrument.

Comparison Pairs	Inter-rater Agreement	Index Instrument	Internal Consistency
	**(Cohen’s Kappa Statistics [S.E.]** ^**a**^ **)**		**(**Cronbach’s *α* **)**
CESD_20_ and K_10_	0.79 (0.073)	CESD_20_	0.93
CESD_20_ and PHQ_9_	0.74 (0.073)	K_10_	0.92
K_10_ and PHQ_9_	0.71 (0.073)	PHQ_9_	0.87

^a^ S.E. = standard error

## Discussion

To our knowledge, this is the first study to examine and compare the diagnostic accuracy and reliability of three common depression screening instruments (CESD_20_, K_10_, and PHQ_9_) and their short forms against a DSM-IV defined gold standard in a HIV-positive population. Overall, each of the screening instruments diagnosed depression with excellent accuracy and reliability. The diagnostic accuracy of the three instruments and their short forms was comparable. Except for the PHQ_2_, each of the instruments showed good-to-excellent sensitivity and specificity, excellent negative predictive value, and moderate positive predictive value at optimal cut-points. The diagnostic accuracy of all instruments may vary according to presence or absence of physical and mental disability. Previously reported somatic symptoms of HIV infection might have affected the diagnostic accuracy of CESD_20_, CESD_10_, and PHQ_2_.

Our results of overall performance are generally consistent with findings previously reported with HIV-positive patients. First, the AUCs and criterion validity statistics of the CESD_20_ and PHQ_9_ were similar to prior findings from HIV-positive patients in Uganda [48]. Although our results were better than the pooled estimates (Se:0.82; Sp:0.73) reported in a recent meta-analysis, substantive between-study heterogeneity was reported in that analysis [22]. Second, the short forms of the three instruments performed comparably, a finding that is consistent with a recent systematic review [21]. Third, as with other studies, most of our test instruments showed moderate rates of false positives when ruling-in for depression [20].

A few differences were noted when we compared our results to the studies conducted in Sub-Saharan Africa. First, unlike Akena et al. (2013) [48], none of the three instruments were diagnostically superior according to AUC values among HIV-positive patients. Additionally, unlike the recent meta-analysis of 113 studies for patients with chronic physical illness, we did not find that the PHQ_9_ was the most sensitive [20]. However, our results of psychometric properties for the PHQ_9_ were generally comparable to that of the general population (Se = 0.88; Sp = 0.88) [23]. Second, the performance of K_10_ in OCS participants was better than previous findings of sensitivity (0.67–0.83) and specificity (0.72–0.77) reported by Akena et al.(2013) and Spies et al.(2009) [48,49]. This may due to systematic differences between the HIV-positive populations in Sub-Saharan Africa and Canada [48,49].

In terms of the optimal cut-points, our results differ from prior findings. For the PHQ9, our optimal cut-point was a total score of 8; previously-reported optimal cut-points have typically been a score of 10 [23,48]. However, results from a recent meta-analysis have shown that cut-points between 8 and 11 all report acceptable diagnostic properties for identifying major depression [50]. For the CESD_20_, our optimal cut-point was slightly higher than those previously reported (i.e., between 16 and 22) among HIV-positive patients [21,22,48], but an optimal point of 23 has also been reported in diabetic populations [51,52]. For the K_10_, our optimal cut-point was within the range reported in prior studies [48,49]. These differences may possibly be due to different criteria that we used when identifying the optimal cut-points. Our optimal cut-points were determined based on three common criteria: 1) diagnostic odds ratios; 2) PROC01; and 3)Youden index. The criteria that were used in prior Sub-Saharan Africa studies focused on maximizing sensitivity and specificity; however, these two measures are only one of the methods to measure the diagnostic accuracy and these criteria may not focus on evaluating predictive performance of a screening instrument.

Our results suggest that shorter instruments are desirable in primary HIV care settings because resource constraints are often found in these settings. Therefore, shorter instruments may find a greater acceptance and yield larger clinical benefits. However, similar to the original screening instruments, the shorter screening instruments also come with moderate positive predictive values, indicating that false positives are likely. We advise that the screening instruments should only be administered when in-depth follow-up assessments are available to properly diagnose depression.

Our results from multivariable ROC analysis indicated that in general, the presence of physical and mental disability may reduce the diagnostic accuracy of screening instruments, thereby making the instruments more difficult to detect depression cases. It is possible that the patients who are eligible for the ODSP programs are sicker and may have more severe physical and mental conditions when compared to other patients who were not eligible for the ODSP program. Similar to prior evidence [20,32], our results may imply that symptoms of chronic conditions may overlap with symptoms of depression especially among patients who have received ODSP subsidies. This would result in an inflation of the total scores for the screening instruments and cause a higher number of false positives, which will lead to a lower PPV to detect depression. As we showed in our further analysis, after we removed some items related to HIV somatic symptoms from the screening instruments, the diagnostic accuracy indicated by the adjusted AUCs were reduced. Therefore, our results suggests that careful consideration must be taken and in-depth follow-up assessments should be available when applying these instruments to patients with chronic illness, especially those with severe physical and mental impairments.

Our study has several strengths. First, this was a multi-center study whose participants may represent typical HIV-positive patients receiving care in Ontario [28]. Second, this is the first study to compare three common screening instruments for depression in a developed country. Unlike Akena et al.(2013) [48], our sample size calculation allowed for detecting differences between AUCs of the instruments, thereby allowing for direct comparison of their diagnostic accuracy. Comparing instruments within a single sample may overcome the heterogeneity issues that have been reported in a recent meta-analysis [21,22]. Third, our analysis also considered the potential impacts of somatic symptoms of HIV infection on the diagnostic accuracy of the instruments [32]. Finally, we adopted advanced statistical techniques to examine the impacts of potential factors that might affect the performance of the instruments [34].

There may be some limitations to our results. First, although the M.I.N.I has frequently been adopted as a “gold standard” for validation studies among the general population and HIV-positive patients [20,22], it is an abbreviated structured interview for psychiatric diagnoses; therefore, it is imperfect when compared to the SCID or ICD-10. This may impact on the discriminatory accuracy of the instruments. However, prior evidence has shown the M.I.N.I to have high sensitivity (94–96%) and specificity (79–88%) for identifying major depressive disorders when compared against SCID or ICD-10 criteria [29–31]. Misclassification from use of the M.I.N.I. as the gold standard would have produced underestimates of sensitivity and specificity. Second, interviewer bias is likely because the M.I.N.I. interviews were conducted by nurses familiar with the clinical histories of their patients. It is possible that the nurses recalled the mental health conditions of their patients from previous appointments and that these recollections affected the interviews. Third, the completion of the screening instruments may have had a positive impact on the performance of the M.I.N.I. through priming (i.e., exposure to the screening instruments may have influenced how participants responded to their M.I.N.I.). This implies that the subsequent M.I.N.I. may have more likely been able to detect depression. Future studies should replicate our results by randomizing the order of the M.I.N.I and the screening instruments to determine if priming is a possibility. Fourth, our study might have been under-powered when testing for equality of AUCs of the instruments because the difference of the AUCs (0.15) that we assumed from Akena et al. (2013) was bigger than that of our current study [48]. Replication with a larger sample is desirable. Fifth, although efforts were made to ensure that our sample represented typical HIV-positive patients in Ontario, differences have been noted between the overall OCS cohort and non-OCS participants [53].

Despite the limitations noted above, our findings demonstrate excellent diagnostic accuracy and reliability of the CESD_20_, K_10_, and PHQ_9_ for current major depression in HIV-positive patients in Ontario. Additionally, the diagnostic accuracy of three instruments and their short forms was comparable. When follow-up assessments become available, shorter instruments may find greater acceptance and yield clinical benefits in relation to depression when incorporated into fast-paced speciality HIV care.
